# Acute Disseminated Toxoplasmosis in Two Specimens of *Macropus rufogriseus* Caused by a Genotype so far Exclusive to South America

**DOI:** 10.3389/fvets.2022.923976

**Published:** 2022-06-15

**Authors:** Luis Fernando Valenzuela-Moreno, María del Carmen Carmona-Muciño, Carlos Cedillo-Peláez, Claudia Patricia Rico-Torres, Héctor Luna-Pastén, María Alejandra Hernández-Rodríguez, Heriberto Caballero-Ortega

**Affiliations:** ^1^Laboratorio de Inmunología Experimental, Instituto Nacional de Pediatría, Insurgentes Sur 3700-C, Colonia Insurgentes-Cuicuilco, Demarcación Territorial Coyoacán, Ciudad de México, Mexico; ^2^Private Zoological Collection, Puebla, Mexico

**Keywords:** *Toxoplasma gondii*, wallabies, parasite burden, qPCR, histopathology, genotyping, Mexico

## Abstract

Macropods are included among the species considered highly susceptible to *Toxoplasma gondii* infection. Clinically, it is difficult to distinguish between acute toxoplasmosis due to primary infection and reactivation of chronic latent infection in susceptible species until pathologic studies are performed. Here, we described the clinical cases and lesions found in two deceased Bennett's wallabies (*Macropus rufogriseus*) with a presumptive diagnosis of toxoplasmosis, as well as the genetic characterization of the *T. gondii* isolates obtained from these specimens. Both animals presented acute infection lesions in the lungs, liver, spleen and lymph nodes associated to *T. gondii* infection. Histopathology and immunohistochemistry also demonstrated tissue cysts of different sizes, indicating that the wallabies were previously infected with this parasite. Two isolates were obtained, one from each specimen and the molecular characterization was done; both isolates were the ToxoDB #116 genotype. This is the first study that reports the isolation of this particular genotype outside South America, and given the histopathological findings, it could be considered virulent for this species. The dynamics of infection that *T. gondii* is causing in definitive and intermediate hosts in a region allows us to know the risks to which the animals and humans that live in the area are exposed, and in the future to implement a preventive medicine plan against this parasite.

## Introduction

*Toxoplasma gondii* is an ubiquitous parasite that can infect virtually any warm-blooded animal, from birds to terrestrial and marine mammals (1). Currently, at least 317 different genotypes have been described worldwide, distributed in 16 haplogroups (2, 3). In zoos, toxoplasmosis is one of the most common causes of death among different species kept in captivity (4). Macropods along with New World primates, lemurs, meerkats, and Pallas cats are included among the species considered to be highly susceptible (4). The most common presentations of toxoplasmosis in macropods are reactivation of a latent infection, usually when they are stressed by transportation or handling, and acute toxoplasmosis due to a virulent strain of *T. gondii* (5). Zoo animals are infected when ingesting oocysts excreted by feral cats or captive wild felids in the collection that can be carried by zoo staff on their boots or washed away contaminating bodies of water, prairies, and enclosures (4, 6). In addition to the diet that is commonly provided to macropods in zoos, these animals can become infected while either grazing or drinking contaminated water with oocysts (7). Clinically, in macropods it is difficult to distinguish between acute toxoplasmosis due to primoinfection and the reactivation of a chronic latent infection (concurrent illness or immunosuppression) in susceptible species until pathologic studies are run (8, 9).

The present study describes the clinical cases and *post mortem* lesions found in two Bennett's wallabies (*Macropus rufogriseus*) with a presumptive diagnosis of toxoplasmosis, as well as the genetic characterization of the *T. gondii* isolates obtained from these specimens. These are the first isolates of this parasite obtained from macropods in México.

## Materials and Methods

### Ethics Approval

This study was supported by the research project with registration number 013/2012 approved by the Reviewing Board of the Instituto Nacional de Pediatría of the Ministry of Health of México (INP; IRB-NIH numbers IRB00008064 and IRB00008065), which includes the Research and Animal Care Committees. The protocol followed national and international regulations for animal welfare and care.

### Clinical Cases

Some wallabies from a colony belonging to a private collection in Puebla, began with various clinical signs such as tachypnoea, diarrhea, hematochezia, and ataxia. *Post mortem* studies were performed in two carcasses and representative samples of organs and tissues were taken. Lung imprints were made with these samples to be observed directly under the microscope. Free and intracellular structures compatible with apicomplexan parasites were visualized, and a presumptive diagnosis of toxoplasmosis was issued. Additionally, lung and liver samples were sent to the Facultad de Medicina Veterinaria y Zootecnia-UNAM Pathology Laboratory to confirm by immunohistochemistry the diagnosis of *T. gondii*. While the samples were processed, the surviving animals were administrated a clinical treatment with Sulfadoxine 200 mg/ Trimethoprim 40 mg/mL (Gorban^®^, MSD Salud Animal) at a dosage of 14.4 mg/kg i.m. every 48 h (15 doses) and Clindamycin 120 mg/mL (Cheminova^®^, Cheminova Salud Animal) 50 mg/kg initial dose and 38.0 mg/kg subsequent doses i.m. once a day for 21 days, and vitamin E + selenium (Selepherol^®^, Vetoquinol) 0.1 mL/kg i.m. every 14 days (three doses). Twelve days after the onset of the treatment, two female marsupials died: wallaby 1 (Wb1) aged 3 years 8 months and wallaby 2 (Wb2) aged 1 year 2 months, which underwent necropsy as previously described (10). Samples of lung, heart, stomach, small intestine, liver, spleen, mesenteric lymph nodes, striated muscle and brain were fixed in 10% buffered formalin for histopathology and immunohistochemistry. Portions of lung, liver, and spleen were kept in refrigeration and used to carry out the molecular diagnosis by endpoint and real-time PCR, and to attempt parasite isolation.

### Cytology

Lung impression smears were made and processed with Romanowsky-type stain (Hemocolorante Rápido^®^, Hycel) The stained slides were observed in an optical microscope (Olympus CH-2; Japan) looking for structures compatible with apicomplexan parasites.

### Histopathology and Immunohistochemistry (IHC)

Representative samples of fixed tissues were processed following the routine histological protocol and stained with hematoxylin and eosin. Immunoreactivity was visualized by the streptavidin-biotin-peroxidase complex (Histostain-Plus, Invitrogen, USA), as previously described with slight modifications (11). Briefly, paraffin-embedded sections were cut and mounted in electrocharged slides (Kling- On- Hier, Biocare); the tissues were deparaffined, hydrated, and blocked following the routine methodology. After that, slides were incubated *T. gondii* positive serum by ELISA, from a mouse experimentally infected with *T. gondii* Me49 strain (1:400 dilution). All slides were washed and incubated with a secondary multispecies biotinylated antibody (Invitrogen, USA), followed by an incubation step with streptavidin-peroxidase. Immunocomplexes were revealed with a commercial solution (Betazoid DAB, Chromogen 3, 3' Diaminobenzidine, Biocare^®^). Sections of liver and spleen of infected mice with *T. gondii* Me49 strain were used as positive controls and for the negative control the primary antibody was replaced with PBS. Histopathology and IHC slides were examined by optical microscopy (Zeiss Axiostar plus; Göttingen, Germany).

### Bioassays in Mice

Bioassays were carried out in a murine model as previously described (12), and the NOM-062-ZOO-1999 guidelines for the handling and care of the mice were followed. Briefly, a portion of lung, liver, and spleen from each wallaby was manually homogenized in sterile PBS with penicillin G (10,000 IU/mL); then two Balb/c mice were inoculated intraperitoneally with 0.5 mL of the homogenate from each tissue. All mice were observed every day and subjected to euthanasia if they showed clinical signs of *T. gondii* infection. Seven days after inoculation a peritoneal lavage was performed using sterile PBS to obtain tachyzoites and reinoculated intraperitoneally in other mice to proliferate the strain. The surviving mice were euthanized 45 days after inoculation; they were considered infected if tachyzoites or tissue cysts were detected in their tissues.

### DNA Extraction

DNA extractions from lung, liver, and spleen from each wallaby as well as from the isolates were performed using Qiagen Gentra^®^ Puregene^®^ Tissue kit (Hilden, Germany), following the manufacturer's instructions. The DNA obtained was quantified using a spectrophotometer (The Thermo Scientific Nanodrop™ 1000, MA, USA) and kept frozen at −20°C until use.

### Molecular Diagnosis and Genotyping

To demonstrate the presence of *T. gondii* in the tissues of the deceased wallabies, endpoint and real time PCR were performed to amplify the 529 bp non-coding repetitive sequence (SeqRep529) and *B1* gene. The parasite burden in the tested tissues was achieved following the methodology described previously (13–16). The isolates obtained from Wb1 and Wb2 as well as the DNA obtained from the lung, liver, and spleen, were genotyped by a multilocus nested PCR assay to amplify nine nuclear and one apicoplast markers (*SAG1*, Alt. *SAG2, SAG3, BTUB, GRA6, c22-8, c29-2, L358, PK1*, and *APICO*). All molecular assays were performed with AmpliTaq Gold™ (Thermo Fisher Scientific, cat. 4311806, MA, USA). The RFLP assays were carried out using specific restriction enzymes (New England, Biolabs^®^, USA) as previously described (17). DNA from RH, Me49, and VEG strains were used as positive and restriction controls (Type I, II, and III, respectively), and sterile water as negative control. RH and Me49 strains were kindly donated by Dr. Rafael Saavedra and VEG strain was purchased from the ATCC (Toxoplasma gondii ATCC^®^ 50861TM). To assure there was no contamination in the nested assays, additional controls were included (positive and negative re-amplification controls).

## Results

### Macroscopic Findings

**Figure 1:**
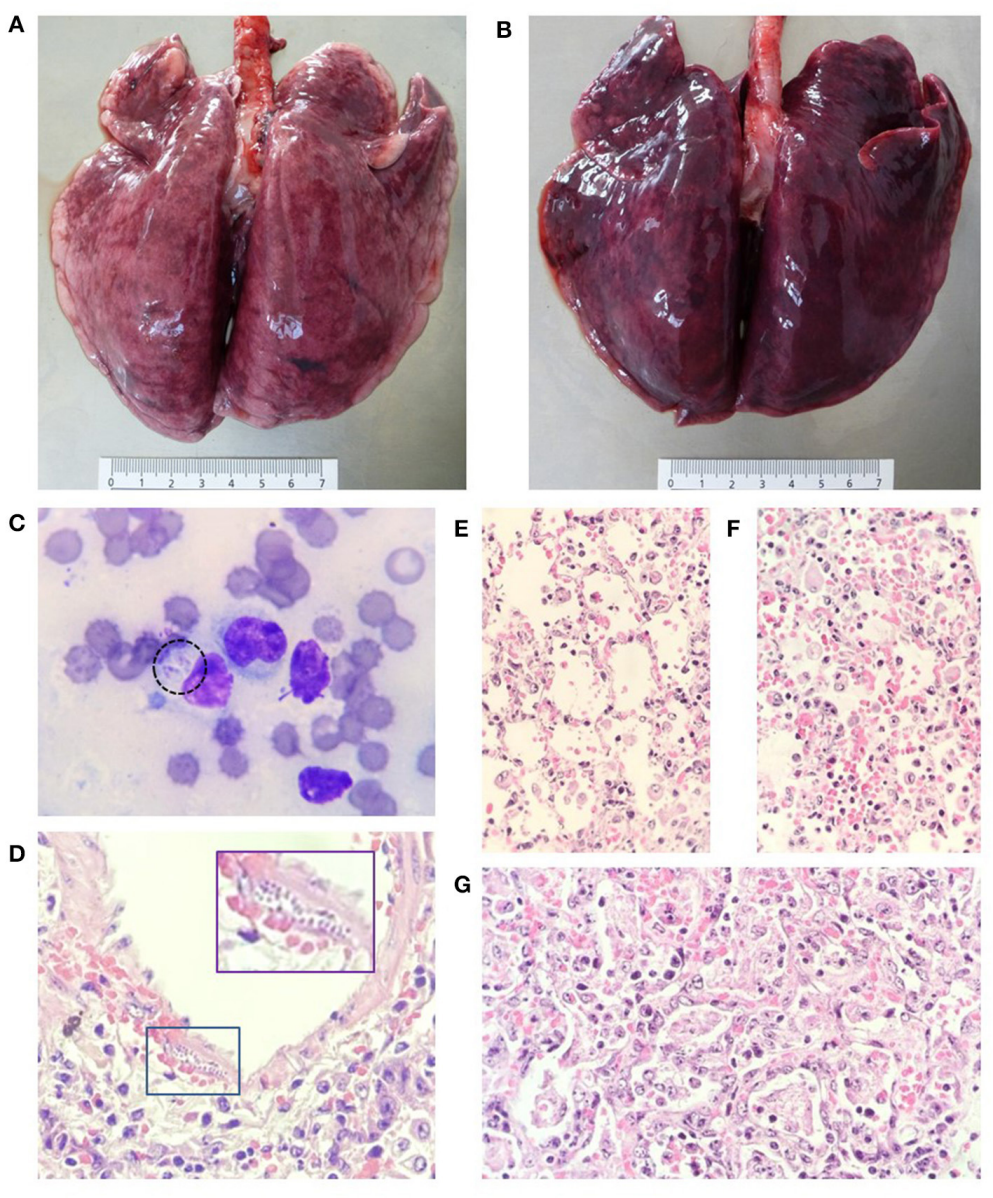
Acute lesions in the respiratory system of both macropods. **(A,B)** Lung lobes with different degrees of interstitial pneumonia and generalized congestion. **(C)** Cytology of lung parenchyma with the presence of alveolar macrophages and two replicating tachyzoites can be seen in the cytoplasm of one of them (dotted circle). **(D–F)** Sections of the lung with different degrees of exudative interstitial pneumonia and the presence of replicating tachyzoites under the muscular layer of a pulmonary artery (box). **(E,F)** Exudative interstitial pneumonia is seen in some areas. **(G)** Other areas with hyaline membrane formation, edema, cellular debris, hemorrhage, and alveolar macrophages in the alveolar lumen. **(C)**: Diff Quick stain, 1000x. **(D–G)**: hematoxylin and eosin 400x.

At necropsy, both wallabies showed good body condition. The nostrils were wet and stained by serosanguineous, foamy fluid which extended throughout the respiratory tract. Lungs failed to collapse, were heavy, diffusely red with multifocal to coalescent pinpoint dark red pattern and had a smooth, wet, shiny surface (Figures 1A,B). The parenchyma had a similar pattern, oozed large amounts of foamy serosanguineous fluid, denoted moderate consolidation and its sections floated irregularly in formalin. Discrete, pale striations were present in the myocardium of Wb1. The digestive tract in both macropods was markedly congested, more prominent at the jejunum and ileum, where the mucosa exhibited moderate, multifocal, up to 0.8 cm erosions and ulcers, partially covered by necro hemorrhagic pseudo membranes. Severe enlargement (up to three times) and moderate superficial and deep hemorrhages were present in the mesenteric lymph nodes. The liver and spleen appeared severely enlarged and congested and the parenchyma was markedly friable. Additionally, the spleen in Wb1 had moderate, pale, punctiform, and intra-parenchymal foci. The kidneys in both animals were markedly congested and the medulla was moderately dark red and eroded at the renal pelvis where few irregular, pale yellow, up to 0.3 long axis concretions were observed. Furthermore, pale striations were present in the renal cortex of Wb2. Marked congestion of the leptomeninges was appreciated in both wallabies.

### Microscopic Findings

From lung imprints, alveolar macrophages were observed and in some of them, parasitic structures compatible with replicating Apicomplexa protozoa were found (Figure 1C). Microscopically, both animals presented acute infection lesions in lungs, liver, spleen, and lymph nodes associated to the infection of *T. gondii*. In pulmonary parenchyma, zones with different degrees of proliferative and exudative interstitial pneumonia were observed, ranging from moderate to severe and composed by pneumocyte proliferation with necrotic zones of the alveolar walls, prevalent mononuclear inflammatory infiltrate and congestion. Alveolar spaces were randomly occupied by mild to severe multizonal edema and exudate composed of alveolar macrophages, neutrophils and lymphocytes, as well as cellular debris and pneumocyte exfoliation, fibrin, and membrane formation. Roundish structures compatible with replicating Apicomplexa parasites were observed within the alveolar wall, alveolar blood vessels and macrophages in mild amounts (Figures 1D–G). The evaluated lymph nodes had the most severe changes, with extensive areas of severe diffuse necrosis and lymphoid depopulation, congestion and moderate multifocal hemorrhages, with abundant amount of intralesional, free and replicating parasitic structures. In contrast, mild to moderate regional lymphoid depopulation prevailed, along with congestive changes and discrete lymphocytic necrosis was observed in the spleen. In the liver, at the periportal level, mixed inflammatory infiltrate was observed with moderate multifocal, mid-zonal necrosis and centrilobular, dissociation of hepatic cords, congestive changes, and random presence of parasitic structures similar to those observed in the lung, but in smaller quantities (Supplementary Figure S1).

**Figure 2:**
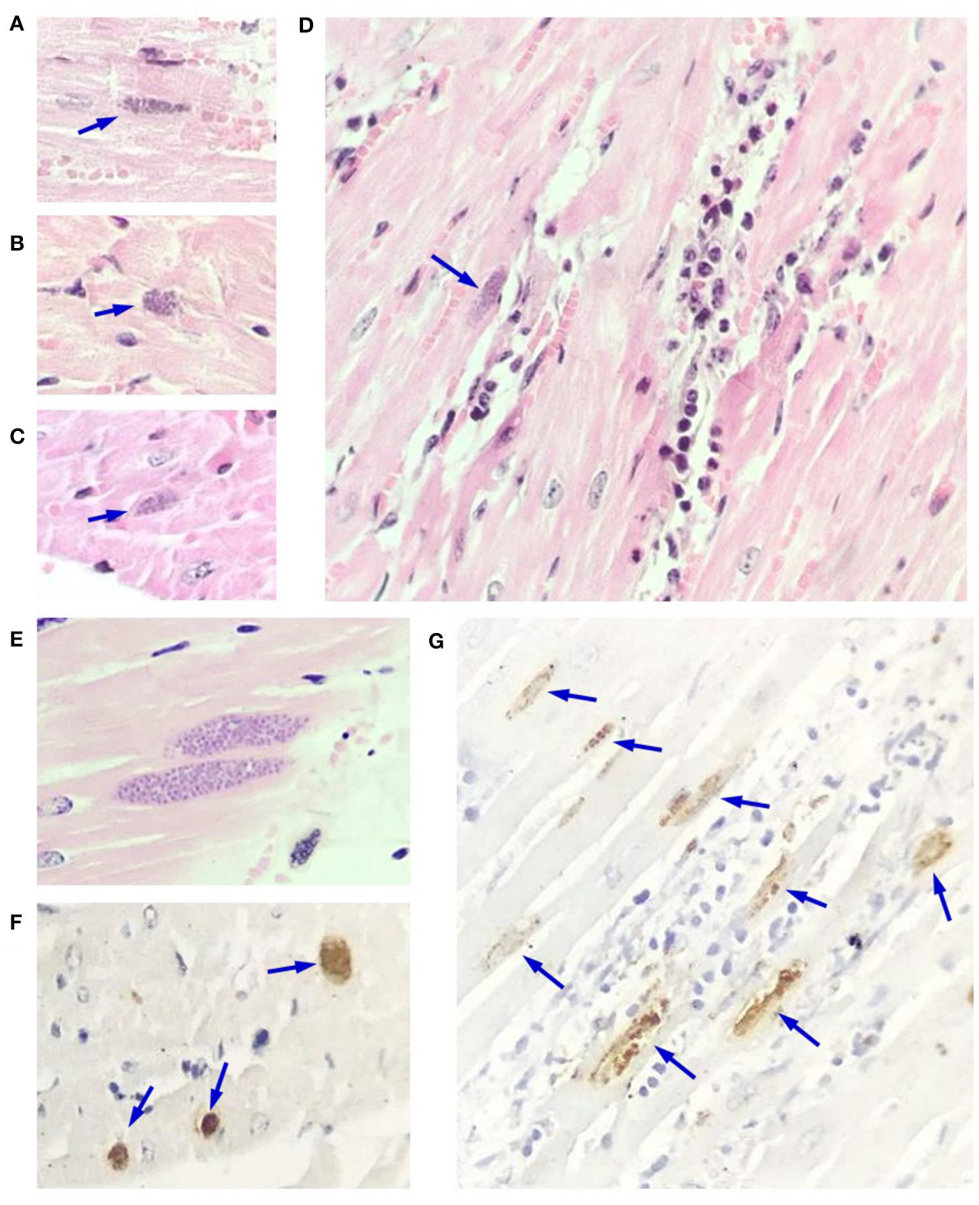
Microscopic findings in myocardium of macropods. **(A–C)** *Toxoplasma gondii* tissue cysts (arrows) of different sizes in cardiac fibers. **(D)** Necrosis and degeneration of fibers, as well as a mixed inflammatory infiltrate with a tissue cyst (arrow) adjacent to the area of injury. **(E)** Myocardial section without alterations with two large tissue cysts. **(F,G)** Myocardial section showing abundant immunopositive tissue cysts with variable marked distribution, without **(F)** and with inflammatory infiltrate **(G)**. **(A–E)**: hematoxylin and eosin, 500x. **(F,G)**: Immunohistochemistry, streptavidin avidin peroxidase complex. **(F,G)** 350x and 480x, respectively.

In myocardium, slight multifocal necrosis of myofibrils, with or without presence of inflammatory infiltrate composed of lymphocytes, plasma cells, and macrophages was observed. These changes were in some areas associated with the presence of tissue cysts of Apicomplexa parasites, while in other regions, the cysts were found without any other alteration. These tissue cysts were of different sizes, with a barely defined wall and some of them with variable tinctorial affinity (Figures 2A–E). Wb1 showed a mild parasitic load, while Wb2 displayed abundant amounts of tissue cysts with up to 15 tissue cysts per 40x field. In addition, mild fibrosis and congestive changes were randomly observed.

In the brain of both animals, a slight degree of neuronal degeneration and necrosis, gliosis, satellitosis, and neuronophagia were observed, predominantly in the cortex and cerebellum. In some of the parenchymal blood vessels as well as choroid plexuses, a random incipient lymphoplasmacytic infiltrate was observed. Sporadically, solitary tissue cysts embedded within the parenchyma were observed, delimited by discrete gliosis and mononuclear inflammatory response (Supplementary Figure S2). The changes displayed by the myocardium and brain are consistent with chronic toxoplasmosis lesions with reactivation and reinfection in the brain due to the presence of free perivascular parasites.

Likewise, sections of the stomach were examined without any pathological alterations, while in the small intestine, only mild to moderate multifocal enteritis was found. In the kidney, several glomeruli presented either membranous glomerulitis or proliferative glomerulitis along with mild to moderate multifocal lymphoplasmacytic interstitial nephritis (figures not shown).

### Immunohistochemistry

Parasites were found in lymph nodes, predominantly seen intralesional (in clusters and free) along the entire tissue. The spleen displayed clusters of immunoreactive parasites were seen in the germinal centers and trabeculae. In the liver, clusters of tachyzoites were found peripheral to the injury sites and immunopositive for anti-*T. gondii* antibodies (Supplementary Figure S1). In the myocardium of both animals, tissue cysts were immunopositive (Figures 2F,G). Also, a low quantity of tissue cysts was observed in the brain. In brain parenchyma and choroid plexuses there was the presence of immunopositive structures suggestive of perivascular free tachyzoites (Supplementary Figure S2). A low number of immunoreactive free parasites were observed in the submucosa of the stomach and in the kidney (figures not shown).

### Molecular Identification, Parasite Load, and Isolates

Five of the six tissues analyzed by molecular biology were positive for the SeqRep529 by conventional PCR. All of them were positive for the *B1* gene by real-time PCR. Wb2 had a higher tachyzoite load in all tissues than Wb1 (up to 2 million parasites per milligram of tissue). The lung was the organ with the highest parasite load in both marsupials, followed by the liver, and spleen. Two isolates were obtained, one from the lung of Wb1 (TgWbMxPue1) and one from the spleen of Wb2 (TgWbMxPue2); both isolates and all tissue samples of the two wallabies were the ToxoDB #116 genotype (Table 1).

**Table 1:** Multiplex multilocus genotyping of *Toxoplasma gondii* strains of infected wallabies from Puebla, México.

**ID**	**Strain denomination**	**Tissue or isolation**	**529 bp repeat PCR**	***B1* gene qPCR**	**Tachyzoites (millions)/mg of tissue**	**Genetic markers**										**ToxoDB PCR-RFLP genotype**
						***SAG1***	***altSAG2***	***SAG3***	***BTUB***	***GRA6***	***c22-8***	***c29-2***	***L358***	***PK1***	***Apico***	
Wb1	TgWbMxPue1^*^	Lung	**+**	**+**	0.26	I	III	III	I	III	II	III	III	III	III	#116
		Liver	**+**	**+**	0.16	I	III	III	I	III	II	III	III	III	III	
		Spleen	**+**	**+**	0.04	I	III	III	I	III	II	III	III	III	III	
		Isolation	**+**	ND	–	I	III	III	I	III	II	III	III	III	III	
Wb2	TgWbMxPue2^*^	Lung	**+**	**+**	2.32	I	III	III	I	III	II	III	III	III	III	#116
		Liver	**–**	**+**	2.16	I	III	III	I	III	II	III	III	III	III	
		Spleen	**+**	**+**	1.39	I	III	III	I	III	II	III	III	III	III	
		Isolation	**+**	ND	–	I	III	III	I	III	II	III	III	III	III	

* *TgWbMxPue1 and TgWbMxPue2 were isolated from lung and spleen, respectively*.

*ND, Not done*.

## Discussion

It is well-known in the scientific literature that Australasian marsupials (including wallabies) are highly susceptible to *Toxoplasma* infection, In this study we describe acute disseminated toxoplasmosis in two wallabies kept in captivity and we show evidence that both of them had a previous infection with a probably different *T. gondii* genotype than the one that caused its death. These marsupials were able to control the primary infection, but that did not counteract the second infection, which was apparently with a more virulent strain for that species.

In México the presence of *T. gondii* in macropods has been scarcely reported. From a retrospective study of paraffin-embedded tissues from various zoos of Mexico, Cedillo-Peláez (11) described cases of disseminated acute toxoplasmosis, reinfections, and reactivations in several macropod species (*M. rufogriseus, M. eugenii, M. rufus* and *M. giganteus*) (11). As in the two cases described here, there were two cases of *M. rufus* in which, in addition to the acute disseminated lesions, tissue cysts of variable size were found in skeletal muscle and brain, although the previous infection did not protect the animals against the second infection that caused their death.

The gross and microscopic lesions found in the two macropods were consistent with those previously reported by others, where the main findings are congestion, interstitial pneumonia, mesenteric lymph-, spleen-, and hepatomegaly (7, 8). Microscopically, the mesenteric lymph nodes were the most damaged tissues, with severe follicular depletion and necrosis of the germinal centers. Some authors have reported spleen atrophy due to necrosis and depopulation like the two animals described here, suggesting that it may be due to a prolonged antigenic stimulation and necrosis caused by parasite replication (5, 7). Unlike the cases of other susceptible species such as squirrel monkeys (*Saimiri sciureus*), which present with minimal or no interstitial pneumonia; in macropods, regardless of the genotype involved, the main findings are interstitial pneumonia with the presence of hepato- and splenomegaly with multifocal necrosis, which suggests that the differences in clinical findings are due to immunological differences between these species (14).

To our knowledge, this is the first time the parasite load is measured in tissues of infected wallabies. Carossino et al. (18) also performed qPCR but only reported mean CT. Supporting the findings we describe, they demonstrated that the lung was the organ with the highest parasite load, followed by the liver as well. The spleen was not included in real-time PCR (18).

It is important to select the correct tissues for the bioassay. In these clinical cases we obtained two isolates from tissues that presented macroscopic lesions and that are reported as organs of acute infection (lung, liver and spleen). Given the clinical signs of the macropods and the laboratory results obtained, we have elements to conclude that the *T. gondii* isolates were responsible of causing the death of both animals. When an acute case is suspected, it is important to process the most affected tissues; otherwise there is a risk of isolating another strain that is latent in chronically infected tissues and not the one that caused the death of the animal (19).

This is the first study to report the isolation of the ToxoDB #116 genotype outside South America. It has been reported in chickens and pigs from Argentina, Brazil, Peru, and Venezuela; thus, this genotype could be disseminated by wild felids distributed throughout the tropical regions of the continent and possibly by migratory birds (20–25). It is worth note that none of the hosts that were infected with this genotype died due to toxoplasmosis, as previously reported (24). However, Clementino Andrade et al. (21) reported that the isolate was virulent in mice (21). It should be noted that the isolate characterized by Rêgo et al. (24) had a 3/3 genotype for *ROP18*/*ROP5*, which is avirulent for mice, while the one described by Clementino Andrade et al. (21) was only typed for the 10 classic markers (21, 24). This is the first time that this genotype is reported in macropods, and, given the histopathological findings, it could be considered virulent for this species. Although the isolate had a majority of type III alleles for the 10 standard markers, and type III alleles are considered to have low virulence in mice, a clonal type III caused disseminated toxoplasmosis and death in *M. rufus, M. rufogriseus* and *M. eugenii* from Argentina and the USA (26, 27).

Even though these two wallabies were treated with Sulfadoxine/Trimethoprim and Clindamycin, they were not effective, and both animals died. Currently none of the treatments described to treat toxoplasmosis in macropods has proven to be effective and the variability of the treatment response could be subject to the specific genotype that caused the clinical signs (8, 27). It has been suggested that the exhibits of macropods and other susceptible species must have a good water drainage, in order to avoid water stagnation and prevent storm water (that could be carrying oocysts) from running into the exhibits (8).

Infectious disease in wildlife kept in captivity are key processes that threaten species conservation. Feral animals are an important factor in the appearance of diseases because they facilitate the transmission of pathogens through contact with wildlife, thus feral populations should be controlled in zoos and private wildlife collections to reduce the possibility of introducing new diseases (28).

In conclusion, ToxoDB #116 genotype of *T. gondii* caused the death of two wallabies from a private collection of marsupials. Macropods are considered susceptible to infection by *T. gondii*, since they did not coevolve with the parasite until the last 300 years, suggesting that their immune system does not control some highly virulent genotypes for this species.

## Data Availability Statement

The original contributions presented in the study are included in the article/Supplementary Material, further inquiries can be directed to the corresponding author/s.

## Ethics Statement

The animal study was reviewed and approved by Reviewing Board of the Instituto Nacional de Pediatría of the Ministry of Health of México (INP; IRB-NIH numbers IRB00008064 and IRB00008065), which includes the Research and Animal Care Committees.

## Author Contributions

LV-M, MC-M, and HC-O: conception or design of this study and data analysis and drafting this article. CC-P, CPR-T, and HC-O: direction and supervision. LV-M, CC-P, CPR-T, HL-P, and MH-R: material acquisition and performed experiments. All authors read and approved the final manuscript.

## Funding

This work was supported by grant 2012/013, Programa E022 Investigación y Desarrollo Tecnológico en Salud from Instituto Nacional de Pediatría, Mexico.

## Conflict of Interest

The authors declare that the research was conducted in the absence of any commercial or financial relationships that could be construed as a potential conflict of interest.

## Publisher's Note

All claims expressed in this article are solely those of the authors and do not necessarily represent those of their affiliated organizations, or those of the publisher, the editors and the reviewers. Any product that may be evaluated in this article, or claim that may be made by its manufacturer, is not guaranteed or endorsed by the publisher.

## Supplementary Material

Supplementary Figure S1 Acute lesions in both macropods. **(A)** Lymph node section with areas of necrosis, hemorrhage, severe lymphoid depopulation, and abundant free and replicating *T. gondii* tachyzoites (arrows) positive for IHC (box). **(B)** Spleen with lymphoid depopulation and presence of replicating tachyzoites (arrow). **(C)** Spleen with abundant *T. gondii* immunoreactive tachyzoites by IHC (arrows). **(D)** Liver with focus of necrosis and mixed inflammatory infiltrate. **(E)** Liver with a cluster of replicating immunoreactive tachyzoites (arrow) close to the area of inflammation detected by IHC. **(A,B,D)**: hematoxylin and eosin, 520x. **(C,E)**: IHC: Immunohistochemistry, streptavidin avidin peroxidase complex, 520x.

Supplementary Figure S2 Microscopic findings in the brain of macropods. **(A,B)** Solitary tissue cysts (arrows) in the midbrain and cerebellum. **(C,D)** Multiple tissue cysts (arrows) in the brain with an inflammatory response and mild gliosis. **(E)** White matter of the middle brain with parenchymal derangement, inflammatory infiltrate and mild gliosis, with the presence of an intralesional tissue cyst (arrow). **(F,G)** Immunopositive tissue cysts from different neuroanatomical regions. **(H–J)** Immunopositive, free and perivascular parasitic structures in brain parenchyma, suggestive of tachyzoites (arrows). **(K–M)** Panoramic view of the choroid plexuses, highlighting two sections (boxes), which when enlarged show immunopositive, free and perivascular parasitic structures, suggestive of tachyzoites [arrows in **(L,M)**]. **(A–E)**: hematoxylin and eosin, **(A–D)**, 500x; **(E)**, 420x. **(F–M)** Immunohistochemistry, streptavidin avidin peroxidase complex. **(F,G)**, 520x; **(H–J)**, 500x; K, 180x; **(L,M)**, 400x.
