# Supplementary figures and images for: Acute Disseminated Toxoplasmosis in Two Specimens of Macropus rufogriseus Caused by a Genotype so far Exclusive to South America
Source: Front Vet Sci. 2022 Jun 15;9:923976. doi: 10.3389/fvets.2022.923976 (PMC9240756; doi:10.3389/fvets.2022.923976)

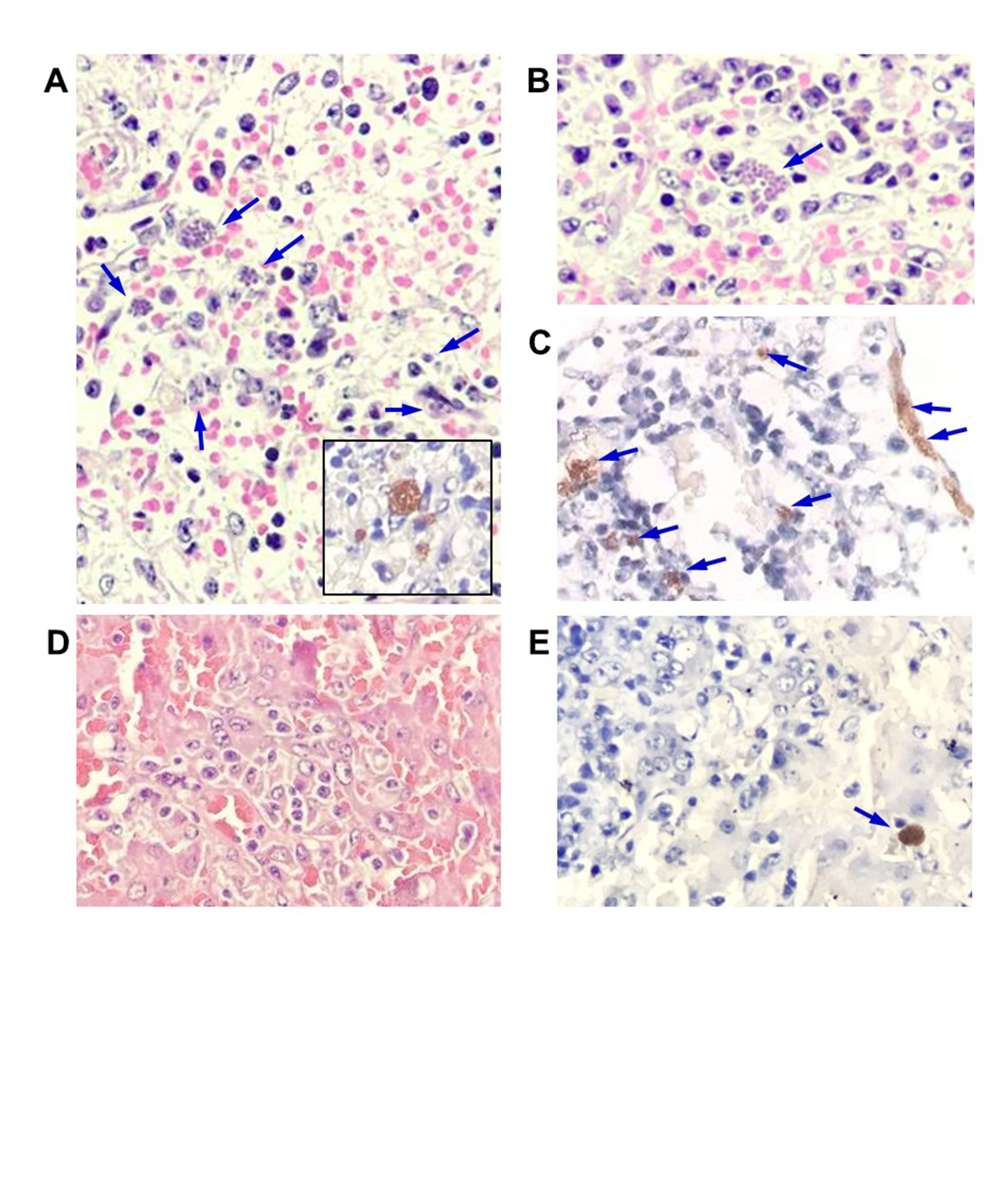

Supplement: Supplementary Figure S1 — Acute lesions in both macropods. (A) Lymph node section with areas of necrosis, hemorrhage, severe lymphoid depopulation, and abundant free and replicating T. gondii tachyzoites (arrows) positive for IHC (box). (B) Spleen with lymphoid depopulation and presence of replicating tachyzoites (arrow). (C) Spleen with abundant T. gondii immunoreactive tachyzoites by IHC (arrows). (D) Liver with focus of necrosis and mixed inflammatory infiltrate. (E) Liver with a cluster of replicating immunoreactive tachyzoites (arrow) close to the area of inflammation detected by IHC. (A,B,D): hematoxylin and eosin, 520x. (C,E): IHC: Immunohistochemistry, streptavidin avidin peroxidase complex, 520x. [file Image_1.jpg]

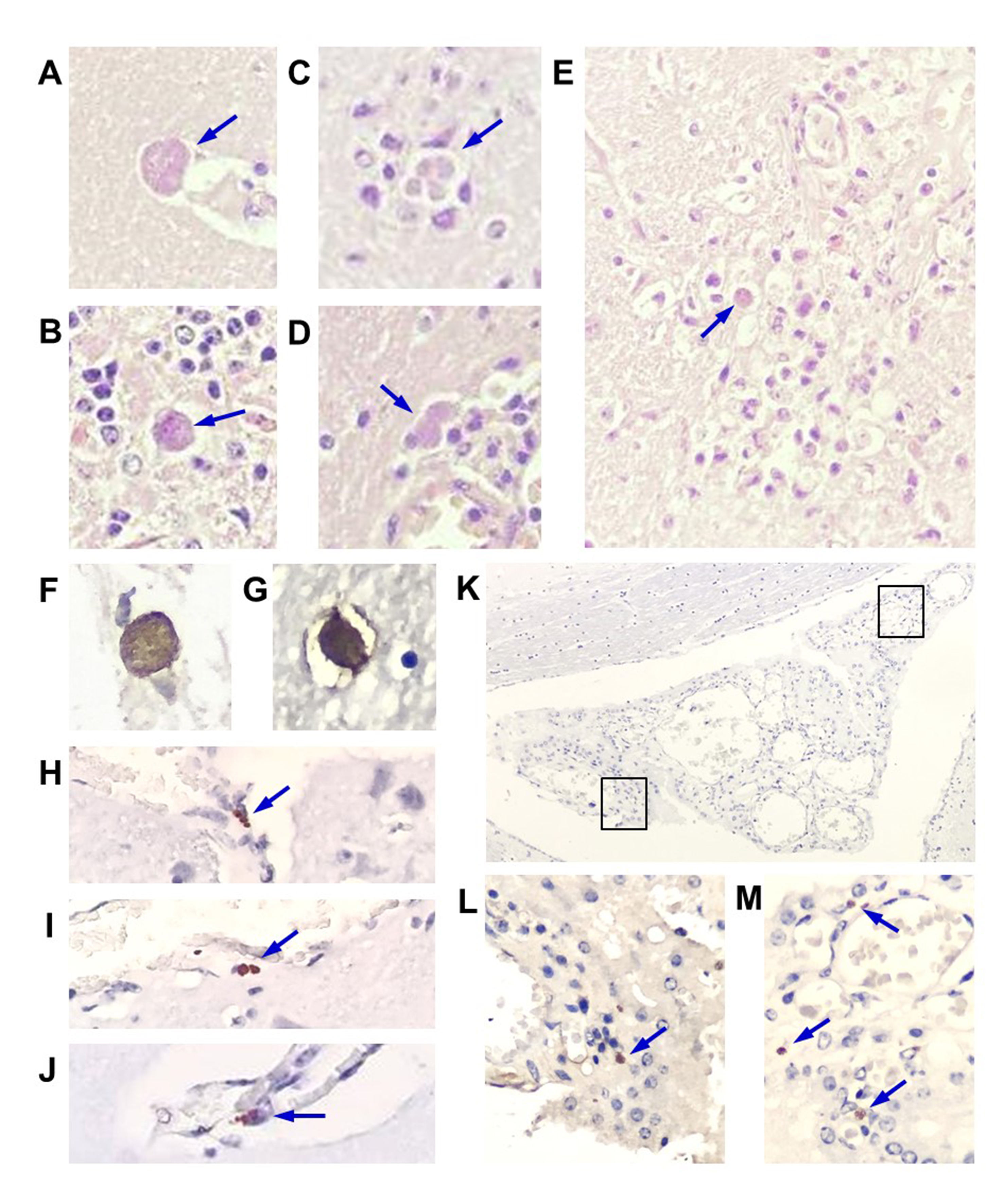

Supplement: Supplementary Figure S2 — Microscopic findings in the brain of macropods. (A,B) Solitary tissue cysts (arrows) in the midbrain and cerebellum. (C,D) Multiple tissue cysts (arrows) in the brain with an inflammatory response and mild gliosis. (E) White matter of the middle brain with parenchymal derangement, inflammatory infiltrate and mild gliosis, with the presence of an intralesional tissue cyst (arrow). (F,G) Immunopositive tissue cysts from different neuroanatomical regions. (H–J) Immunopositive, free and perivascular parasitic structures in brain parenchyma, suggestive of tachyzoites (arrows). (K–M) Panoramic view of the choroid plexuses, highlighting two sections (boxes), which when enlarged show immunopositive, free and perivascular parasitic structures, suggestive of tachyzoites [arrows in (L,M)]. (A–E): hematoxylin and eosin, (A–D), 500x; (E), 420x. (F–M) Immunohistochemistry, streptavidin avidin peroxidase complex. (F,G), 520x; (H–J), 500x; K, 180x; (L,M), 400x. [file Image_2.jpg]
